# Widespread natural selection on metabolite levels in humans

**DOI:** 10.1101/gr.278756.123

**Published:** 2024-08

**Authors:** Yanina Timasheva, Kaido Lepik, Orsolya Liska, Balázs Papp, Zoltán Kutalik

**Affiliations:** 1 Institute of Biochemistry and Genetics, Ufa Federal Research Center of Russian Academy of Sciences, 450054 Ufa, Russia;; 2 Department of Medical Genetics, Bashkir State Medical University, 450008 Ufa, Russia;; 3 Department of Computational Biology, University of Lausanne, CH-1015 Lausanne, Switzerland;; 4 Center for Primary Care and Public Health, University of Lausanne, CH-1010 Lausanne, Switzerland;; 5 Swiss Institute of Bioinformatics, CH-1015 Lausanne, Switzerland;; 6 HCEMM-BRC Metabolic Systems Biology Lab, H-6726 Szeged, Hungary;; 7 Synthetic and Systems Biology Unit, National Laboratory of Biotechnology, Institute of Biochemistry, Biological Research Centre, HUN-REN, H-6726 Szeged, Hungary;; 8 Doctoral School of Biology, University of Szeged, H-6726 Szeged, Hungary;; 9 National Laboratory for Health Security, Institute of Biochemistry, Biological Research Centre, HUN-REN, H-6726 Szeged, Hungary

## Abstract

Natural selection acts ubiquitously on complex human traits, predominantly constraining the occurrence of extreme phenotypes (stabilizing selection). These constraints propagate to DNA sequence variants associated with traits under selection. The genetic signatures of such evolutionary events can thus be detected via combining effect size estimates from genetic association studies and the corresponding allele frequencies. Although this approach has been successfully applied to high-level traits, the prevalence and mode of selection acting on molecular traits remain poorly understood. Here, we estimate the action of natural selection on genetic variants associated with metabolite levels, an important layer of molecular traits. By leveraging summary statistics of published genome-wide association studies with large sample sizes, we find strong evidence of stabilizing selection for 15 out of 97 plasma metabolites, with nonessential amino acids displaying especially strong selection signatures. Mendelian randomization analysis reveals that metabolites under stronger stabilizing selection display larger effects on a range of clinically relevant complex traits, suggesting that maintaining a disease-free profile may be an important source of selective constraints on the metabolome. Metabolites under strong stabilizing selection in humans are also more conserved in their concentrations among diverse mammalian species, suggesting shared selective forces across micro- and macroevolutionary timescales. Overall, this study demonstrates that variation in metabolite levels among humans is frequently shaped by natural selection and this may act through their causal impact on disease susceptibility.

Human metabolites provide a unique insight into metabolic pathways underlying health and disease and can serve as a useful tool for precision medicine with multiple applications, including the discovery of new therapeutic targets and the development of novel protocols for diagnostics or monitoring the progression of the disease and the efficacy of treatment (Zhang et al. 2015). Recent advances in metabolomic research have identified a number of biochemical processes involved in the pathogenesis of complex diseases, such as cancer, atherosclerosis, and diabetes (Jin and Ma 2021).

Intermediate metabolites can also help to elucidate the influence of natural selection owing to the evolutionary advantages and disadvantages resulting from the ability of living organisms to produce compounds with functions beneficial or detrimental for fitness. Although dysregulation of several specific metabolites has been linked to human diseases, potentially indicating strong stabilizing selection to preserve their levels, the direction and strength of natural selection shaping metabolite levels are generally unknown. Several lines of observations suggest that stabilizing selection on metabolite levels might be prevalent. First, evolutionarily distant species show substantial similarities in metabolite levels, indicating widespread evolutionary conservation of the metabolome (Ma et al. 2015; Park et al. 2016). Second, the levels of central metabolites obey simple optimality principles, indicating that metabolite levels might represent optimal values (Tepper et al. 2013). However, not all metabolites are expected to be under equally strong stabilizing selection, and there might be larger room for selectively neutral alterations for some metabolites than for others. Indeed, a recent multispecies comparison revealed wide differences in the extent of conservation of individual metabolite concentrations during evolution, likely driven by differing amounts of functional constraints across metabolites (Liska et al. 2023). Furthermore, a remarkable acceleration of metabolome evolution has been reported in the human lineage compared with other primates, potentially indicating the action of directional selection on specific metabolites (Bozek et al. 2014). However, the general patterns of selection shaping human metabolite concentrations remain essentially unknown.

Evidence for stabilizing selection acting on a particular trait can be inferred from the relationship between the multivariable effect size (*b*) and minor allele frequency (MAF) of genetic variants responsible for its regulation (Zeng et al. 2018). The observed omnigenic architecture of complex traits suggests that a large number of trait-associated genetic variants have a very small effect, whereas only a few of them have larger effects. Individuals carrying alleles associated with larger (detrimental) effects on a trait under strong (stabilizing) selection will have a higher chance to have decreased fitness and hence will tend to be purged from the gene pool. This results in a decreased allele frequency. It is generally viewed that most traits under stabilizing selection have an optimal value for fitness, and individuals with larger deviations in either direction tend to be selected out with increasing probability. Hence, it is reasonable to assume that in such a case there is an inverse relationship between the squared effect size and MAF (or the variability of the genotype, i.e., 2 × MAF × (1 − MAF)). Stronger selection leads to the sharper decline in MAF upon increase in effect size. Therefore, it has been proposed to estimate selection strength acting on a phenotype, denoted by α, as the value that best fits the *b*^2^∼ [2 × MAF × (1 − MAF)]^α^ relationship for the given phenotype (Schoech et al. 2019).

More importantly, simulation studies demonstrated that negative α values point to stabilizing, whereas positive *α* values point to either directional or disruptive selection (Zeng et al. 2018). For typical complex traits, a reasonable *α* was estimated to be around −0.25 (Speed and Balding 2019). It was shown that stratifying heritability models for functional annotations, LD scores, and MAF can improve heritability estimation and trait prediction (Speed et al. 2020). Hence, identifying the signatures of stabilizing selection is important both for understanding the genetic underpinnings of phenotypic variation and for understanding evolution.

Such methodology has not yet been applied to molecular traits, such as metabolite concentrations, owing to the lack of statistical power because of the unavailability of a sufficiently large sample size. The recent emergence of genome-wide association summary statistics for metabolites (Lotta et al. 2021) provided the first such opportunity. To leverage the newly available data, we reliably estimated selection strength for 97 out of 135 metabolites from a wide range of biochemical classes. First, we compared the performances of weighted and unweighted LDAK-Alpha and BLD-LDAK+Alpha models using summary statistics data for 59 complex traits available from the UK Biobank. We then applied the weighted model to obtain the selection strength signatures for 135 metabolites using the summary statistics data generated in a cross-platform meta-analysis of genetic effects on levels of blood metabolites measured in large-scale population-based studies (Lotta et al. 2021). We also investigated the causal relationship between metabolites and 51 clinically important complex traits using Mendelian randomization (MR) in order to explore the relationship between the selection strength estimates for the studied metabolites and their impact on clinically relevant complex traits. Finally, we used an orthogonal measure of evolutionary constraints by comparing cross-species metabolite concentrations among mammals, and compared it with the strength for stabilizing selection obtained from human GWAS data.

## Results

### Confirmation of the heritability model using UK Biobank data for complex traits

We first applied the BLD-LDAK + Alpha model implemented in the SumHer functionality of the LDAK software to 59 complex traits available in the UK Biobank (Supplemental Table S1; Supplemental Fig. S1). After testing different heritability models, we established that fitting the 65-parameter BLD-LDAK + Alpha model for the majority of the 59 studied traits led to *α* estimates within the range of −0.9 to −0.15. Out of the 59 traits, 25 were also tested in a previous study using raw genotype data (Zeng et al. 2018). For these 25 overlapping traits, we observed reasonable similarity (Supplemental Fig. S2) between selection strength estimates by the 65-parameter BLD-LDAK + Alpha model versus approaches based on raw genetic data: *r* = 0.31 (Zeng et al. 2018) and *r* = 0.39 (Schoech et al. 2019). These observed concordance values (*r* = 0.31 and *r* = 0.39) tend to be higher than the agreement between the selection estimates from Zeng et al. versus Schoech et al. (*r* = −0.26) (Supplemental Fig. S2), although not significantly so.

### Metabolome-wide signatures of natural selection

**Figure 1.**
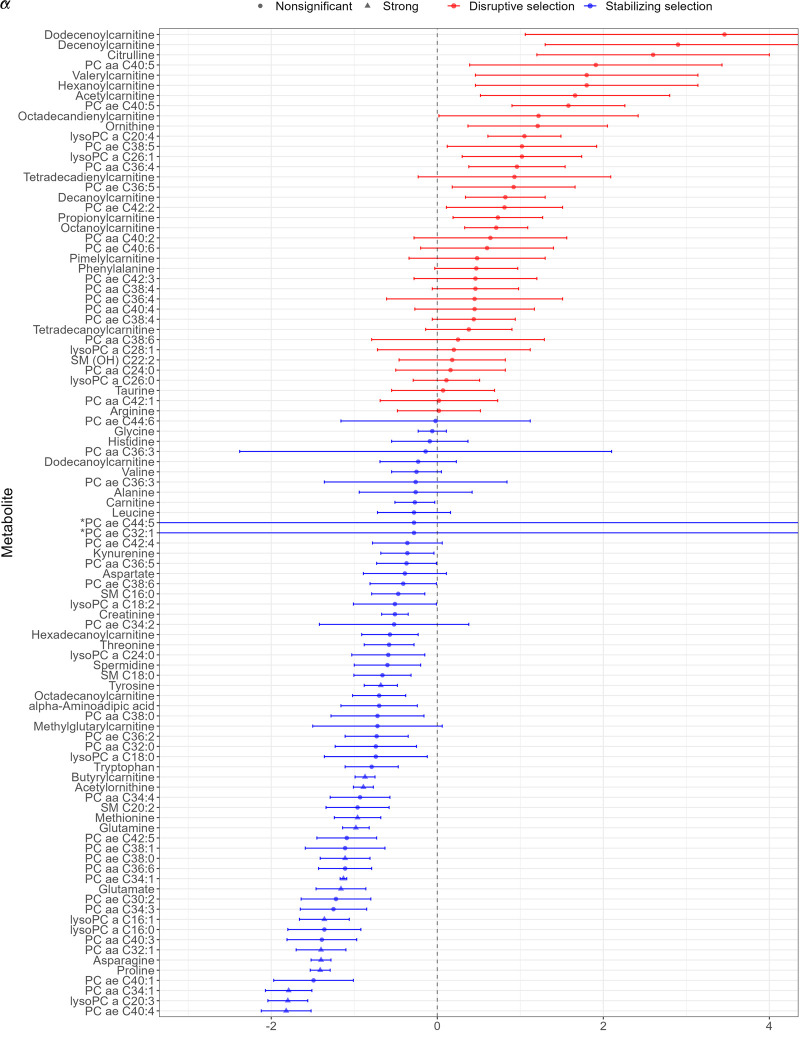
Metabolites with selection estimates showing stabilizing selection ($\hat{\alpha }$ < 0) and disruptive selection ($\hat{\alpha }$ > 0). Circle symbols represent nonsignificant selection estimates; triangles refer to metabolites with a selection strength *P*-value surviving multiple testing correction (*P* < 0.05/97). Error bars represent SEs. Metabolites showing exceptionally large SEs are indicated by an asterisk, such as PC ae C32:1 (SE = 98.5) and PC ae C44:5 (SE = 86.1).

Having tested the BLD-LDAK + Alpha model on complex traits, we performed the analysis to explore, for the first time, the evidence of selection for 135 metabolites for which summary statistics are available from GWAS with large sample sizes (Lotta et al. 2021). Notably, 38 metabolites did not produce stable maximum likelihood estimates for the selection parameter (decided based on visual inspection of the profile likelihoods), most likely owing to low heritability, incompatible genetic architecture, or small sample size. Out of the remaining 97 metabolites (with *α* estimates ranging from −1.82 to 3.43), 66 led to selection estimates not significantly different from zero. Twenty-eight metabolites with nominally significant (*P* < 0.05) selection estimates showed stabilizing selection ($\hat{\alpha }$ < 0), and three are estimated to be under directional or disruptive selection ($\hat{\alpha }$ > 0). Figure 1 illustrates the estimated selection strength values for these metabolites.

We have found strong evidence (*P* < 0.05/97) of stabilizing selection for 15 metabolites (tyrosine, butyrylcarnitine, acetylornithine, methionine, glutamine, PC ae C38:0, glutamate, proline, PC ae C34:1, lysoPC a C16:1, PC aa C32:1, asparagine, PC aa C34:1, lysoPC a C20:3, PC ae C40:4). These estimates were robust with a smooth profile likelihood function (Supplemental Fig. S3). Evidence for the three nominally significant metabolites under disruptive/directional selection (citrulline, lysoPC a C20:4, PC ae C40:5) did not reach the adjusted statistical significance level (*P* < 0.05/97) (Fig. 1).

**Figure 2.**
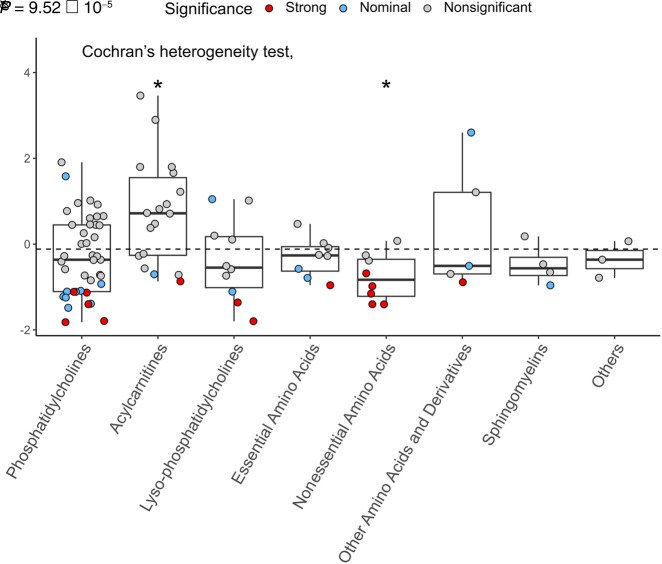
Selection estimates vary significantly across metabolite classes. Plot compares the estimated α values across different metabolite compound classes. Note that the groups essential amino acids and nonessential amino acids include proteinogenic amino acids, and other amino acids and derivatives include nonproteinogenic amino acids and compounds derived from them. The $\hat{\alpha }$ values show significant heterogeneity across metabolite classes (adjusted Cochran's heterogeneity test, *Q* = 31.95, *P* = 9.52 × 10^−5^). Comparisons of metabolites within a particular class against the rest of metabolites reveal two classes that differ significantly (denoted by asterisks): acylcarnitines and nonessential amino acids (adjusted *t*-test *P* = 0.044 and *P* = 0.0038, respectively). Boxes depict the interquartile range (IQR); the solid black line shows the median; and the whiskers extend to 1.5 IQR. The dashed line indicates average $\hat{\alpha }$ value across all investigated metabolites. Colors represent the significance level of the selection estimates.

Metabolites showing strong evidence for stabilizing selection span several major compound classes, such as amino acids and derivatives, phosphatidylcholines (PCs), and lysophosphatidylcholines (lysoPCs). This is despite the highly variable detection power favoring amino acids, which have the highest GWAS sample size. To investigate whether selection differs across major metabolite classes, we focused on estimated α values, which are unaffected by detection power. We found significant heterogeneity in α estimates across metabolite groups (modified Cochran's *Q* = 31.95, *P* = 9.52 × 10^−5^) (Fig. 2). In particular, nutritionally nonessential proteinogenic amino acids (NEAAs) display significantly lower $\hat{\alpha }$ values than the rest of the metabolites, whereas no such trend was observed for nutritionally essential amino acids (EAAs; modified *t*-test *P* = 0.0038 and *P* = 0.496, respectively). This pattern indicates that amino acids that are both needed for protein synthesis and synthesized by the human body are under especially strong stabilizing selection. Conversely, acylcarnitines tend to have positive selection coefficients (average $\hat{\alpha }$ value = 0.85) significantly higher than those of the rest of the metabolites (*P* = 0.044). This suggests that they might be under less stringent evolutionary constraint (either no or disruptive/directional selection).

### Selection strength correlates with cross-species evolutionary conservation of metabolite levels

The selective forces shaping human metabolism are likely to be shared, at least partly, among mammalian species. If so, metabolites that are under stronger stabilizing selection in human populations are expected to be more evolutionarily conserved in their concentrations over macroevolutionary timescales. To test this hypothesis, we used a recent approach to infer a score that captures the extent of evolutionary conservation of metabolite concentrations for individual metabolites based on cross-species comparisons (Liska et al. 2023). In brief, this metabolite conservation score is based on the Brownian motion model of trait evolution. The rate parameter of the Brownian motion model provides a simple and robust measure of the effective rate of evolution for quantitative traits, even if the actual evolutionary process departs from Brownian motion (Ackerly 2009). The conservation score is defined as the inverse of this rate parameter (Methods). Note that the conservation score has been shown to capture variation in functional constraints across metabolites (Liska et al. 2023).

**Figure 3.**
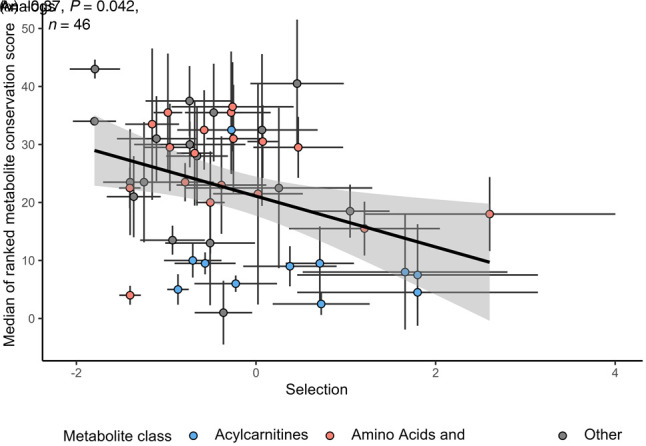
Metabolites under stronger stabilizing selection show stronger evolutionary conservation across mammals. Pearson correlation coefficient is indicated on the plot. The indicated *P*-value is determined by adjusting for nonindependence between the metabolites (see Methods). Evolutionary conservation of metabolite concentrations is estimated by a single “metabolite conservation score” calculated from cross-species metabolomic data in four organs: brain, heart, liver, and kidney (see Methods). Line depicts the fitted linear regression. Colors represent two metabolite classes, amino acids (including essential, nonessential, and other amino acids) and their derivatives (red) and acylcarnitines (blue). Error bars represent the SE of the $\hat{\alpha }$ (*x*-axis) and SD of the metabolite ranks across the four organs.

We focused on a metabolomic data set containing the relative concentrations of 262 metabolites in four major organs of 26 mammalian species, spanning an evolutionary period of ∼200 million years (Ma et al. 2015), and calculated an aggregated score of metabolite conservation across the four organs (see Methods). Out of the 97 metabolites with $\hat{\alpha }$ value, 46 were present in the cross-species data set, including several amino acids, phosphatidylcholines, and acylcarnitines (Supplemental Table S2). In line with our expectations, metabolites with lower $\hat{\alpha }$ values (i.e., stronger stabilizing selection) tend to have higher cross-species conservation scores (*R* = −0.37, *P* = 0.042) (Fig. 3). This effect is largely because of the higher conservation of metabolites with negative $\hat{\alpha }$ values than those with positive $\hat{\alpha }$ values. This trend indicates that metabolites under stabilizing selection in humans, as detected from GWAS, tend to evolve especially slowly on macroevolutionary timescales. For example, amino acids frequently show strong negative $\hat{\alpha }$ values and also tend to be highly conserved among mammalian species, whereas acylcarnitines often display positive $\hat{\alpha }$ values and are among the least conserved metabolites (Fig. 3). The presence of this trend is all the more remarkable as cross-species evolution also involves adaptive shifts in metabolite levels outside the human lineage (Ma et al. 2015) that likely diminish the above correlation. Furthermore, the correlation might be also diminished by the fact that we calculated conservation scores from tissue metabolome data owing to the lack of appropriate cross-species data on plasma metabolite levels. A leave-one-out analysis shows that the correlation is not driven by conservation scores of a particular organ (Supplemental Table S4).

### Linking selection strength with the causal effect on clinically relevant traits

Natural selection to maintain the optimum values of clinically important complex traits might underlie the strong signatures of stabilizing selection on specific metabolites. To test this hypothesis, we first used a MR approach to unveil the causal links between metabolites and health-related traits. Applying an inverse-variance-weighted MR method (Burgess et al. 2013), we estimated the causal effects of the 97 metabolites on 51 complex traits with clinical relevance (Supplemental Table S2), including lipids/proteins, blood cell type composition, and adiposity, anthropometric, cardiovascular, metabolic, hormonal, cognitive, and psychiatric traits. Note that to minimize the overlap between the metabolites and the complex traits, we removed all causal effects that were larger than 0.5 in absolute value (e.g., the causal effect of creatinine [as metabolite] on creatinine [as complex trait] was estimated to be 0.989).

**Figure 4.**
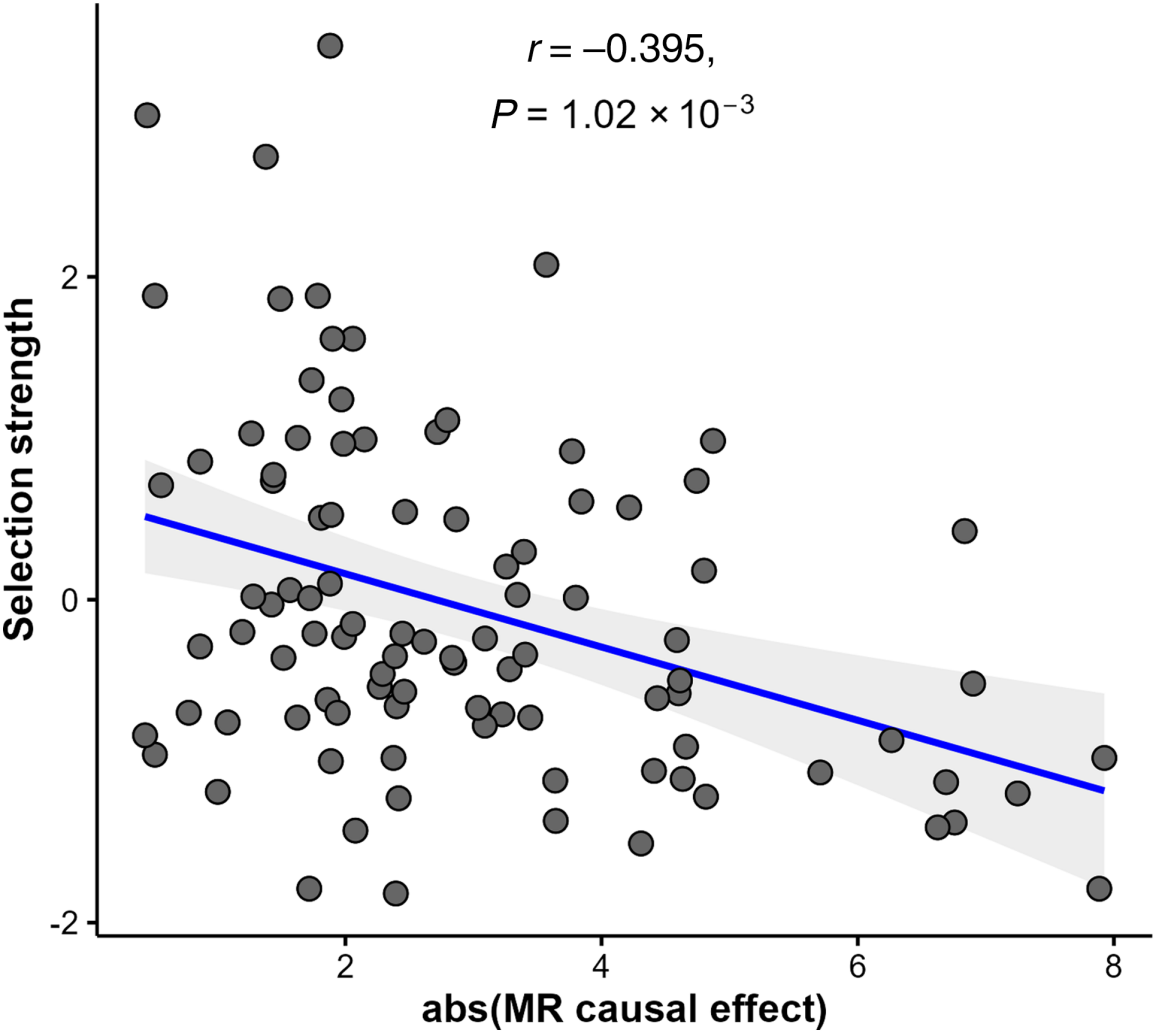
The relationship between metabolite importance (measured as total absolute causal effect size on 51 complex traits) and selection strength for 97 metabolites with genetic instruments.

We then interrogated the relationship between the selection strength estimates for the 97 metabolites and their absolute MR effect sizes on these 51 traits. This analysis yielded negative correlations for 48 out of the 51 tested traits (Supplemental Fig. S4; Supplemental Table S3), meaning that metabolites under stronger stabilizing selection show larger effect sizes on complex, clinically relevant traits. In total, 23 of the 51 traits survive multiple testing correction, controlling the false-discovery rate at 5% (using the Benjamini–Hochberg step-up procedure). For example, we observed a rather strong relationship (*r* < −0.4, *P* < 3.3 × 10^−4^) for glucose, glycated hemoglobin, clinically measured serum urate, IGF1, testosterone, and SHBG. Milder correlations were observed with various high-level traits, such as basal metabolic rate (*r* = −0.28, *P* = 0.01), pulse rate (*r* = −0.31, *P* = 0.0066), and triglycerides (*r* = −0.28, *P* = 0.01). Naturally, individual metabolites are not expected to be constrained through selection on individual medical traits; hence, we do not expect to observe very strong correlations. Therefore, to quantify the overall importance of each metabolite, we calculated the total absolute effect of each metabolite on the 51 studied traits combined. These importance scores were then compared with the previously derived selection strength estimates (see Fig. 4) and revealed a very robust negative trend (*r* = −0.395, *P* = 1.02 × 10^−3^), confirming the observation that metabolites with a more prominent impact on a range of complex clinical phenotypes are bound to be under stronger stabilizing selection.

## Discussion

We performed the first study, to our knowledge, to assess the stabilizing/disruptive selection strength of metabolites using summary statistics from metabolomic GWAS. Leveraging this approach, we were able to estimate stabilizing selection strength for 97 metabolites, via investigating the allele frequency-dependent genetic architecture of metabolomic traits. Information on the strength of selection can be useful in assessing the role of the metabolites as diagnostic, prognostic, or treatment-response biomarkers.

To tackle these questions, we first established that the 65-parameter LD score–weighted BLD-LDAK+Alpha model produces the most robust estimates of selection strength for complex traits, and hence, applied it to 135 metabolites with available GWAS summary statistics. Our findings indicate that the majority of the studied amino acids, PCs, lysoPCs, acetylcarnitines, and related compounds with evidence for selection display negative $\hat{\alpha }$ values indicative of stabilizing selection, with only three metabolites showing nominally significant positive $\hat{\alpha }$. Our results align with the view that the majority of complex traits are under stabilizing selection that eliminates metabolite-associated genetic variants from the population to avoid deleterious fitness effects (Koch and Sunyaev 2021). Although it is unsurprising that high-level complex phenotypes, such as reproductive or cardiovascular traits, generally show negative *α* estimates (Zeng et al. 2018; Schoech et al. 2019; Speed et al. 2020; Zeng et al. 2021) owing to their close links to fitness, our work expands this notion to molecular traits. Alterations in molecular traits might not necessarily influence higher-level phenotypes and fitness, and therefore, it has been proposed that such traits are more likely to evolve neutrally (Zhang 2018). Our work demonstrates that metabolites, an important layer of molecular traits, also show strong signatures of stabilizing selection in their genetic architectures, albeit less frequently than high-level complex traits (Zeng et al. 2018).

Compared with the selection strength profiles of high-level phenotypes identified in our study, the *α* values for the metabolites tend to vary wider, and because of the relatively smaller GWAS size available, the estimates are noisier (cf. Fig. 1 and Supplemental Fig. S1). Recent technological advancements allowed the detection of hundreds of metabolic compounds that can potentially be used as biomarkers for diseases or drug targets. However, different operating standards and the lack of reference values obtained from healthy subjects lead to large discrepancies in detected metabolite levels across different platforms and laboratories, impeding metabolic profiling (Saigusa et al. 2021) and downstream analyses, like ours. The MR analysis results demonstrate that many metabolites have causal effects on clinically relevant, complex traits, although, in line with transcriptome-wide MR analyses (Porcu et al. 2019), these effects are minor. Still, we hypothesized that clinically more important metabolites would have a larger impact on common disease traits. At the same time, if small changes in metabolites lead to disease consequences, their levels are expected to be under stronger stabilizing selection. This prompted us to check whether larger (absolute) causal disease effects couple with stronger (negative) selection values, and we found that selection strength estimates negatively correlated with the absolute causal effect of these metabolites for almost 95% of the 51 tested traits. These observed trends, even if not always strictly statistically significant, in combination provide clear evidence that metabolites with a larger impact on clinically important outcomes tend to be under stronger selection. Our results also imply that maintaining various clinically relevant traits close to their optimal values is an important source of evolutionary constraints on certain metabolites.

We found significant variation in the estimated strength of selection across major metabolite classes. Notably, nutritionally NEAAs showed especially strong stabilizing selection, whereas nutritionally EAAs displayed no such pattern. Both classes of amino acids serve as building blocks for protein biosynthesis and play crucial roles in various other cellular processes (Wu et al. 2014). However, although EAAs must be supplied by the diet to sustain life, NEAAs can also be synthesized by the human body. We speculate that as the availability of food sources can change rapidly, human cells have evolved a greater tolerance for fluctuations in EAA levels compared with NEAA. This tolerance likely ensures a sustained capacity for protein synthesis and proper cellular function even with fluctuating dietary sources and, as a consequence, allows more neutral changes in the levels of EAAs during evolution. Maintaining stable NEAA levels may be important for another reason, beyond their role in protein synthesis. Natural selection may act to prevent excessive NEAA synthesis, which would be energetically wasteful and divert resources from other important metabolic processes. Clearly, further work is needed to decipher the cellular mechanisms explaining the strong stabilizing selection on NEAA levels.

In addition to NEAAs, we also detected signatures of stabilizing selection among PCs, lysoPCs, and acylcarnitines. LysoPC a C16:1 ($\hat{\alpha }$ = −1.37) was associated with type 2 diabetes; lysoPC a C20:3 ($\hat{\alpha }$ = −1.79), with blood pressure level (Ha et al. 2012; Huang et al. 2022). PC aa C32:1 ($\hat{\alpha }$ = −1.41) and methionine were identified as one of the key links between homocysteine pathway and telomere length (van der Spek et al. 2019). Butyrylcarnitine, a short-chain acylcarnitine (C4) and the only member of the acylcarnitine family with a stabilizing selection strength estimate ($\hat{\alpha }$ = −0.84), was identified as an informative prognostic marker for neonatal hypoxic-ischemic encephalopathy: a condition characterized by hypoxia triggering a complex response that leads to energy failure, disruption of cellular homeostasis, morphologic changes in microglial cells, and mitochondrial failure (López-Suárez et al. 2019). Note, however, that as metabolite heritability increases, not only do we obtain lower SEs (i.e., improved estimator precision) but also stabilizing selection estimates become slightly stronger (Supplemental Fig. S5). This might mean that metabolites whose level is under stronger constraint are less impacted by the environment and under stronger genetic control.

Our analyses indicate that certain metabolites might have been shaped by adaptive evolution in the recent evolutionary past of humans. First, we found three metabolites (citrulline, lysoPC a C20:4, PC ae C40:5) with nominally significant positive $\hat{\alpha }$. Second, we examined whether some large-effect genetic variants may have violated the LDAK model assumptions to yield positive selection coefficient, but did not find any evidence for these metabolites having particularly large-effect mQTLs. In such cases, the variants associated with the metabolite are under positive selection, potentially indicating disruptive or directional selection on the metabolite concentration (Zeng et al. 2018). Directional selection may simply reflect ongoing selection, whereby the population mean has not yet reached the trait optimum. Disruptive selection could emerge owing to underdominance (Hartl and Clark 1989) or could be driven by the pervasive pleiotropy we observe for common variants (Jordan et al. 2019). The latter would reflect antagonistic pleiotropy when the same allele (or two alleles in strong linkage disequilibrium) may be beneficial for one trait but detrimental for another. The strongest positive $\hat{\alpha }$ was detected for citrulline, the key intermediate of the urea cycle, involved also in nitric oxide production (Aguayo et al. 2021). Clearly, future studies on larger sample sizes should provide further evidence that these metabolites are under directional/disruptive selection and are not simply under relaxed selection.

We found a remarkable agreement between our estimates of selection based on GWAS data and patterns of metabolite concentration divergence over longer evolutionary timescales. Specifically, we found that metabolites with lower $\hat{\alpha }$ values tend to be more conserved in the concentrations across diverse mammalian species. The observed association remains significant (*P* = 0.0238) even if we replace the genetic correlation matrix with the phenotypic correlation of the metabolite levels from the Ma et al. (2015) study. This finding has at least two general implications. First, it is broadly consistent with the neutral theory of molecular evolution positing that most within-species polymorphisms and between-species divergences at the molecular level are effectively neutral, that is, permitted rather than favored by natural selection (Kimura 1983). Although the theory was originally proposed to explain DNA and protein sequence evolution, it could in principle apply to complex molecular traits as well (Zhang 2018). Second, the agreement between $\hat{\alpha }$ and between-species conservation score suggests that the selective constraints preserving metabolite levels are at least partly shared between human and other mammalian species, including distantly related taxonomic groups. A recent study suggests that variation in evolutionary conservation across metabolites can be explained by a simple model in which natural selection preserves flux through key metabolic reactions while permitting the accumulation of selectively neutral changes in enzyme activities (Liska et al. 2023). Future works should test the extent to which this general model explains stabilizing selection on human metabolite levels.

Last, our results have implications for the understanding of the genetic architecture of molecular traits. Although for nonmolecular traits no $\hat{\alpha }$ parameter was observed to go below −1, we here report some metabolites showing more extreme selection strength. This threshold has a special meaning, because $\hat{\alpha }$ < −1 indicates that low-frequency markers have more per-SNP-heritability than common ones, whereas *α* > −1 points to an architecture in which the average contribution of a common SNP to the trait heritability is more than that of the rare counterparts. This implicates that as opposed to complex traits, for metabolites, the rare variant contribution may be far more important. Note that the $\hat{\alpha }$ values smaller than −1 need to be interpreted with care: Theory and forward simulations show that the *α* model is a good fit to the relationship of β^2^ and 2*p*(1 − *p*) expected if data were generated based on the Eyre-Walker model for a wide range of parameter values. However, as the selection parameter, *S*, of the Eyre-Walker model tends to infinity (strongest selection), the corresponding *α* value approaches −1. Therefore, $\hat{\alpha }$ value lower than −1 cannot be produced by data generated by the Eyre-Walker model. Hence, such observed values mean either that the Eyre-Walker model is not compatible with the observed data or that the simple *α*-model is not a good fit for too high *S* values.

Our study has several methodological limitations that can influence results interpretation. Variable sample size and heritability of the studied trait are key factors determining the power of the study to detect the selection strength acting on a trait. Therefore, our analyses cannot be used to establish a priority ranking but rather to identify some metabolites with significant evidence for selection. The interpretation of the *α* parameter requires care because values below −1 are difficult to translate to classical evolutionary models. More broadly, the *α* parameter may reflect direct selection on the metabolite itself as well as indirect selection acting on genetically correlated traits. Consequently, the observed stabilizing selection on metabolites might represent “apparent stabilizing selection” rather than necessarily reflecting a direct impact on organismal fitness (McGuigan et al. 2011). Additionally, our estimates for *α* and metabolite-to-trait causal effects assume that the input GWAS summary statistics have been controlled for population stratification, assortative mating, parental/dynastic effects, etc. Furthermore, comparisons between different noisy estimates (such as selection strength, evolutionary conservation score, causal effect of metabolites on complex traits) lend themselves to regression dilution bias and, hence, underestimated correlations and low statistical power. Finally, we used the largest available metabolomic GWAS data set, but the results obtained in our study require further validation using independent study samples and different quantification methods.

## Methods

We analyzed genome-wide summary statistics for 135 metabolites, including amino acids, biogenic amines, acylcarnitines, lysoPCs, PCs, sphingomyelins, and hexoses, made available recently from a large (up to 86,507 individuals) genome-wide meta-analysis study (Lotta et al. 2021).

### Stabilizing selection

We used the SumHer approach implemented in the LDAK software (Zhang et al. 2021), having compared the performance of different heritability models to identify the optimal version for assessing the selection strength. The tested models were based on the LDAK model in which the expected per-SNP-heritability of a SNP depends on both the LD and MAF:

$$E[b_{j}^{2}|\;f_{j},\omega_{j}]=\frac{h^{2}}{M}\omega_{j}{\left[2\;f_{j}(1-\;f_{j})\right]}^{\alpha },$$

where $b_{j}^{2}$ is the multivariable squared effect size of SNP *j*, *f*_*j*_ is the observed MAF, *h*^2^ is the trait heritability, *M* is the number of causal markers, and ω_*j*_ represents the SNP weights, which are inversely proportional to the LD score (computed based on the local LD structure). Note that *b*_*j*_s are the theoretical multivariable SNP effects that are the starting point of the model. Through mathematical derivations (similar to those in the LD-score regression) the marginal GWAS effect sizes can be expressed (including the LD score as a multiplier owing to LD dilution). Parameter *α* represents the strength of stabilizing selection. This parameter is estimated from the data via profile likelihood maximization. In brief, we fixed the selection strength parameter (*α*) and maximized the likelihood function for *h*^2^. This procedure was repeated for 31 values of *α*, ranging from −1 to 0.5 with a step size of 0.05. Finally, we plotted the maximum likelihood values against the value of *α* and fitted a quadratic polynomial to these points. Once the estimates for the coefficients of the polynomial were established, the maximum and the negative Hessian (of its second derivative with respect to *α*) were computed, yielding the maximum likelihood estimator for *α* and its variance. For some traits, we observed that the optimal *α* values fall outside of the [−1, 0.5] range, and in such situations, we extended the range to either [−2.0, 1.0] or [−1.0, 4.0] as necessary.

We adjusted this procedure to use more complex, stratified heritability estimation with 65 genomic annotations (resulting in stratum-specific heritability estimates). This BLD-LDAK+Alpha model extends the LDAK+Alpha model by adding 65 annotations provided by Hujoel et al. (2019). We tried simpler models (without annotation, or equal weights [ω_*j*_ = 1]) and compared the performance of these models using the summary statistics data for 59 well-studied complex traits (including height, BMI, SBP, etc.) from the UK Biobank (N = 361,194). The estimates for *α* from the BLD-LDAK+Alpha model came closest to the ones reported in previous studies (Zeng et al. 2018; Speed et al. 2020); therefore, this latter model was applied to investigate the selection signatures for 135 metabolites (with the sample size varying between 9363 and 86,507) (Lotta et al. 2021).

### MR analysis

We applied an inverse-variance-weighted MR approach (Burgess et al. 2013) to explore the causal relationship between metabolites and 51 complex traits with clinical relevance (Supplemental Table S2), including lipids and adiposity, cardio, metabolic, and cognitive traits, using genetic variants associated with the metabolites as instrumental variables. The magnitude of the obtained causal effects was contrasted to the selection strength estimates for the studied metabolites to test whether stabilizing selection is acting stronger on metabolites with more important effects on clinically relevant phenotypes.

### Estimated evolutionary conservation scores of metabolites in mammals

To assess the evolutionary conservation of metabolite levels in mammals, we used a previous cross-species metabolomic study that quantified the relative concentrations of more than 250 metabolites in four organs (brain, heart, kidney, and liver) of 26 mammalian species (Ma et al. 2015). To calculate metabolite conservation scores (i.e., the extent to which the concentration of any given metabolite is permitted to change over the course of evolution), we fit a Brownian motion model of trait evolution on each metabolite in each organ across the phylogeny of 26 species. We defined the conservation score of each metabolite as the inverse of the rate of concentration change that was inferred from the phylogeny. To get a unified conservation score for each metabolite across the four organs, we first imputed all missing values (i.e., metabolites that were not measured in all four organs) by calculating the median conservation score of the given metabolite across the measured organs. Then, we calculated the ranks of metabolite conservation scores across the metabolome in each organ and used the median rank value across the four organs as an aggregate measure of metabolite conservation. Only metabolites measured in more than one organ were included in this analysis, providing us with aggregate conservation scores for a total of 249 metabolites, out of which 46 were among the 97 metabolites for which selection strength could be estimated.

### Correlations between metabolites

In all our analyses, we accounted for the pairwise correlations between the level of these metabolites in the human population. We chose to use genetic correlations over phenotypic correlations for two reasons. First, estimating stabilizing selection strength based on human GWAS data is solely based on the genetic part of each metabolite. Two metabolites with the exact same genetic basis but with different environmental components would have identical stabilizing selection estimates according to the applied genetic approach (even if the true selection parameters might be different). The same holds for the MR analyses: MR estimates are purely based on the genetic components of the exposure and the outcome; hence, they do not change if we modify the exposure with a nongenetic factor. Second, phenotypic correlations are available only for a fraction (up to 46 out of 97) of the metabolites, which would massively reduce our power. Therefore, we computed pairwise genetic covariances between every pair of these 97 metabolites using cross-trait LD-score regression (Bulik-Sullivan et al. 2015). This software also returns the corresponding estimator variances. First, off-diagonal elements of this correlation matrix that were not even nominally significantly different from zero (*P* > 0.05) were set to zero. Next, because of estimation errors, this matrix is not positively semidefinite; hence, we applied weighted “bending,” a technique to make minor adjustments (average absolute change in the covariance values being 0.007) to the covariance matrix to achieve this property (Jorjani et al. 2003). The bending weights were set to be inversely proportional to the variances of the covariance estimates. The bent covariance matrix was then transformed to a correlation matrix.

### Association tests allowing for the correlation between metabolites

To compute the linear association between certain quantities (cross-species vs. human selection or human selection vs. causal effects) while accounting for this cross-metabolite correlation (Σ), we used random effect linear regression applied to the scaled (zero mean and unit variance) quantities: selection *α*, conservation scores, MR causal effect estimates. The model y∼r⋅x+ϵ with $\epsilon ∼N(0,\sigma^{2}\cdot \Sigma )$ was used to estimate *r*. Although the $\hat{r}=(y^{\prime }\cdot x)/(x^{\prime }\cdot x)$ estimator remains unbiased, its variance changes to $Var(\hat{r})=\;\sigma^{2}\cdot \frac{x^{\prime }\cdot \Sigma \cdot x}{{\left(x^{\prime }\cdot x\right)}^{2}}$. This is a much more robust solution than the likelihood maximization owing to numerical instability caused by several very small eigenvalues of the Σ matrix.

### Detecting heterogeneity between selection strength estimates across eight metabolite classes

To quantify heterogeneity, we started off with the distribution of the estimated selection strength values

$$\hat{\alpha }∼N(0,\Omega )$$

with $\Omega \;:=SE(\hat{\alpha })\cdot \Sigma \cdot SE(\hat{\alpha })$. Let $\hat{\alpha_{i}}$ denote the mean selection value in metabolite class *i = 1*…*K* and $\bar{\alpha }$ represent the overall mean across all classes. If we define $v_{j}^{\left(i\right)}=\frac{1}{n_{i}}$ when metabolite *j* is in class *i* and zero otherwise with *n*_*i*_ as the number of metabolites in class *i*, we then have $\hat{\alpha_{i}}={v^{\left(i\right)}}^{\prime }\cdot \hat{\alpha }$. The joint distribution of class-specific means is

$$\left(\begin{array}{c} \hat{\alpha_{1}}\\ \vdots \\ \hat{\alpha_{K}} \end{array}\right)∼N(\bar{\alpha },W),$$

where

$$W_{i,j}:={v^{\left(i\right)}}^{\prime }\cdot \Omega \cdot v^{\left(j\right)}.$$

Therefore, we define our modified Cochran's heterogeneity *Q*-statistic as

$$Q:={\left(\begin{array}{c} \hat{\alpha_{1}}-\bar{\alpha }\\ \vdots \\ \hat{\alpha_{K}}-\bar{\alpha } \end{array}\right)}^{\prime }\cdot W\cdot \left(\begin{array}{c} \hat{\alpha_{1}}-\bar{\alpha }\\ \vdots \\ \hat{\alpha_{K}}-\bar{\alpha } \end{array}\right)∼\chi_{K-1}^{2}.$$

Similarly, to detect significant differences between mean selection value of a class against all other metabolites, we can use the same trick by defining $w_{j}^{i}=1/n_{i}$ when metabolite *j* is in class *i* and $w_{j}^{i}=-1/n_{-i}$, where *n*_−*i*_ is the number of metabolites not in class *i*. This way under the null,

$$t:=w^{i^{\prime }}\cdot \hat{\alpha }∼N(0,w^{i^{\prime }}\cdot \Omega \cdot w^{i}),$$

which allows us to assign a *P*-value to the test of the mean selection in class *i* being the same as outside class *i*.

## Supplementary Material

Supplement 1

Supplement 2

Supplement 3

Supplement 4

Supplement 5
