# Supplementary material for: Widespread natural selection on metabolite levels in humans
Source: Genome Res. 2024 Aug;34(8):1121–9. doi: 10.1101/gr.278756.123 (PMC11444169; doi:10.1101/gr.278756.123)
Supplement: Supplement 1 [file Supplemental_Figures.pdf]

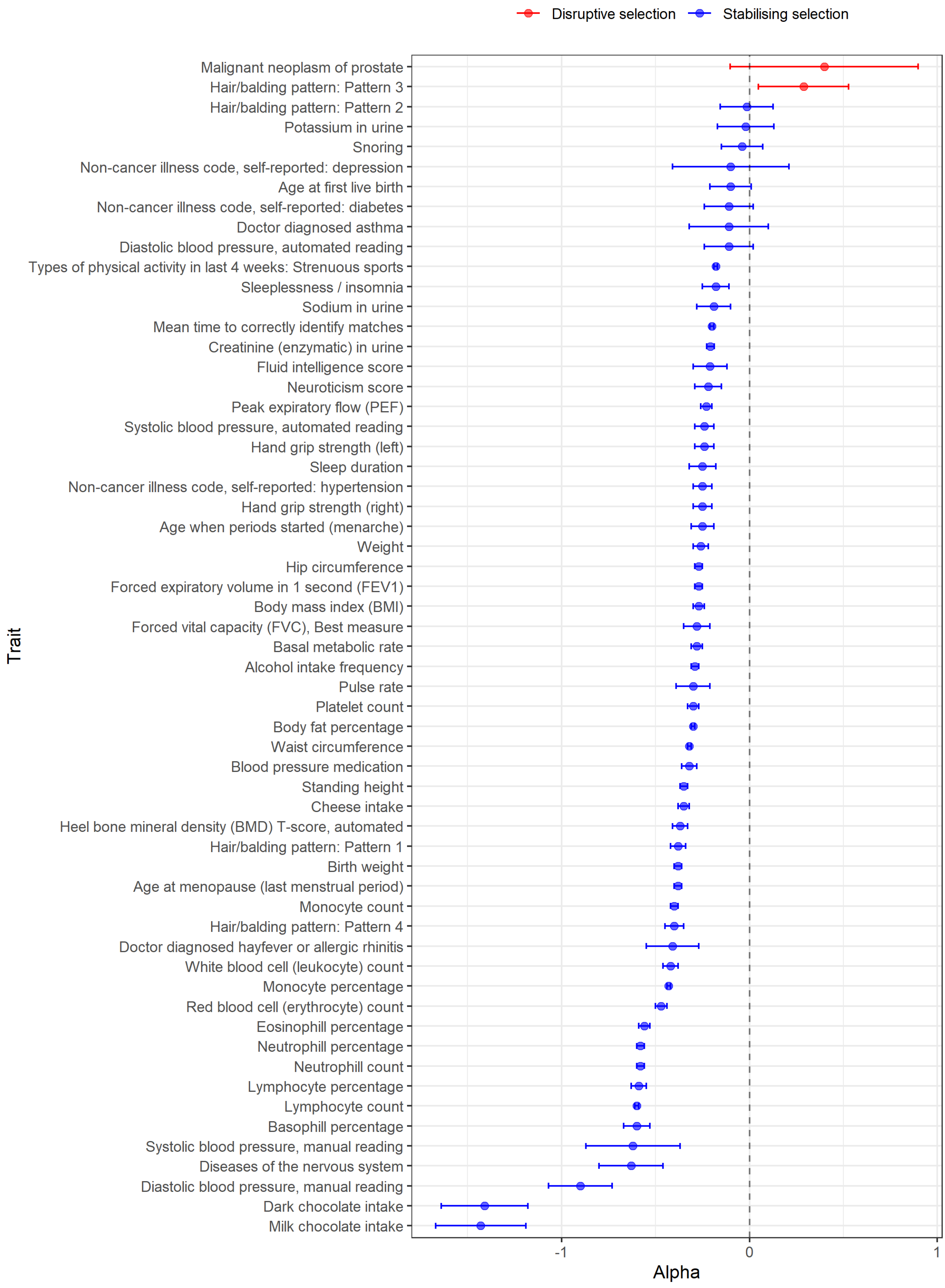

Supplementary Figure S1. Selection strength estimates for the 59 complex traits, available from the UK Biobank

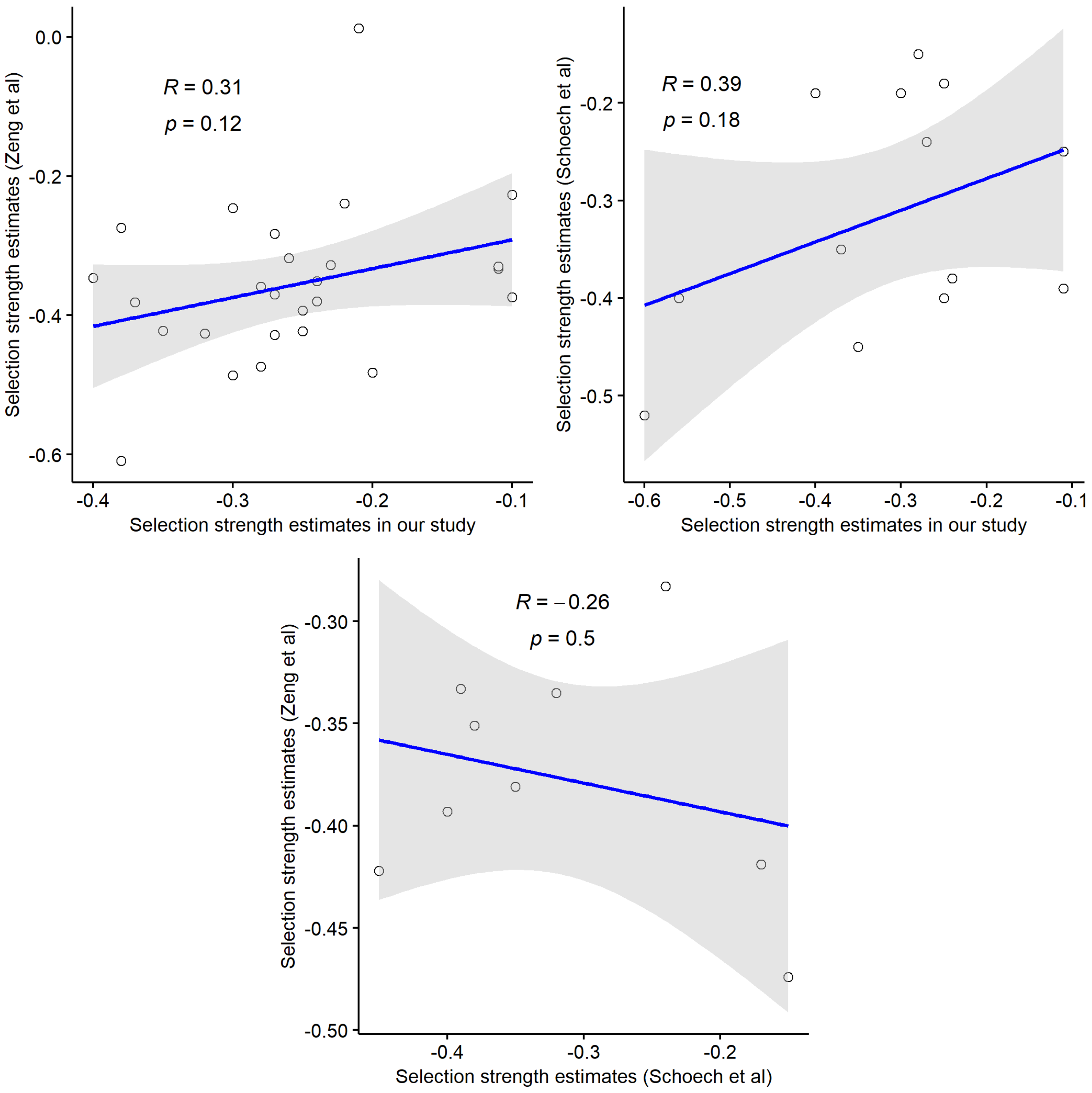

Supplementary Figure S2. The comparison of the selection strength estimates obtained in our study and in the previous studies(Zeng et al. 2018, Schoech et al. 2019)

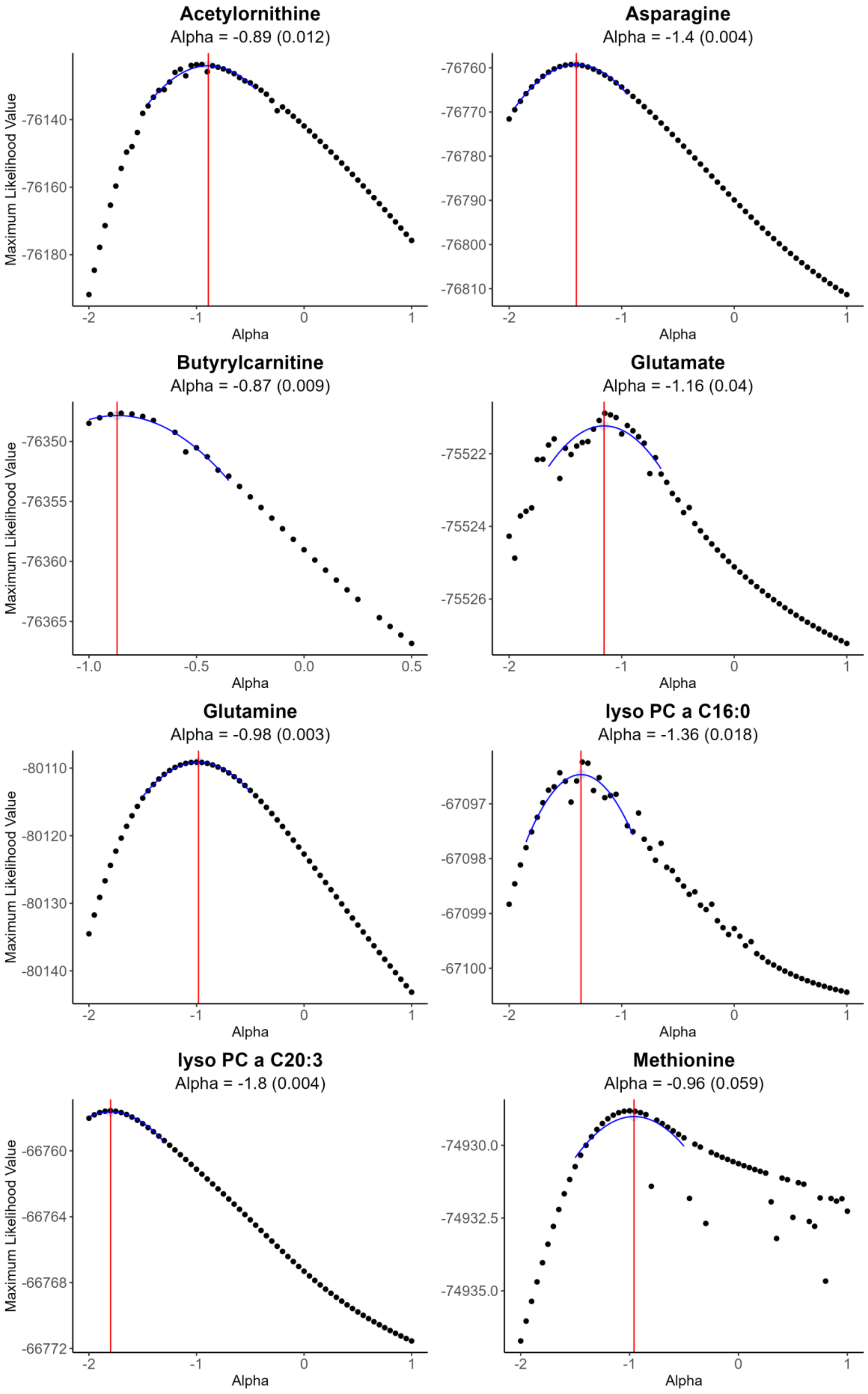

Supplementary Figure S3. Profile likelihood plots for the metabolites with the selection strength estimates significantly different from 0. On these plots we can observe two kinds

of unusual behaviours: (a) noisy likelihood estimates leading to an overall wiggly likelihood profile (e.g. lysoPC a C16:1) and (b) some outliers in an otherwise smooth likelihood surface. The former situation may reflect a general difficulty optimizing the likelihood function (for most alpha values) and hence more unreliable. However, multiple optimisations (for different alpha values) inform each other and the fitted curve is more robust and reduces this noise. The second situation represents a sporadic maximization failure and we omitted these points when fitting the likelihood function (Methionine falls into this category).

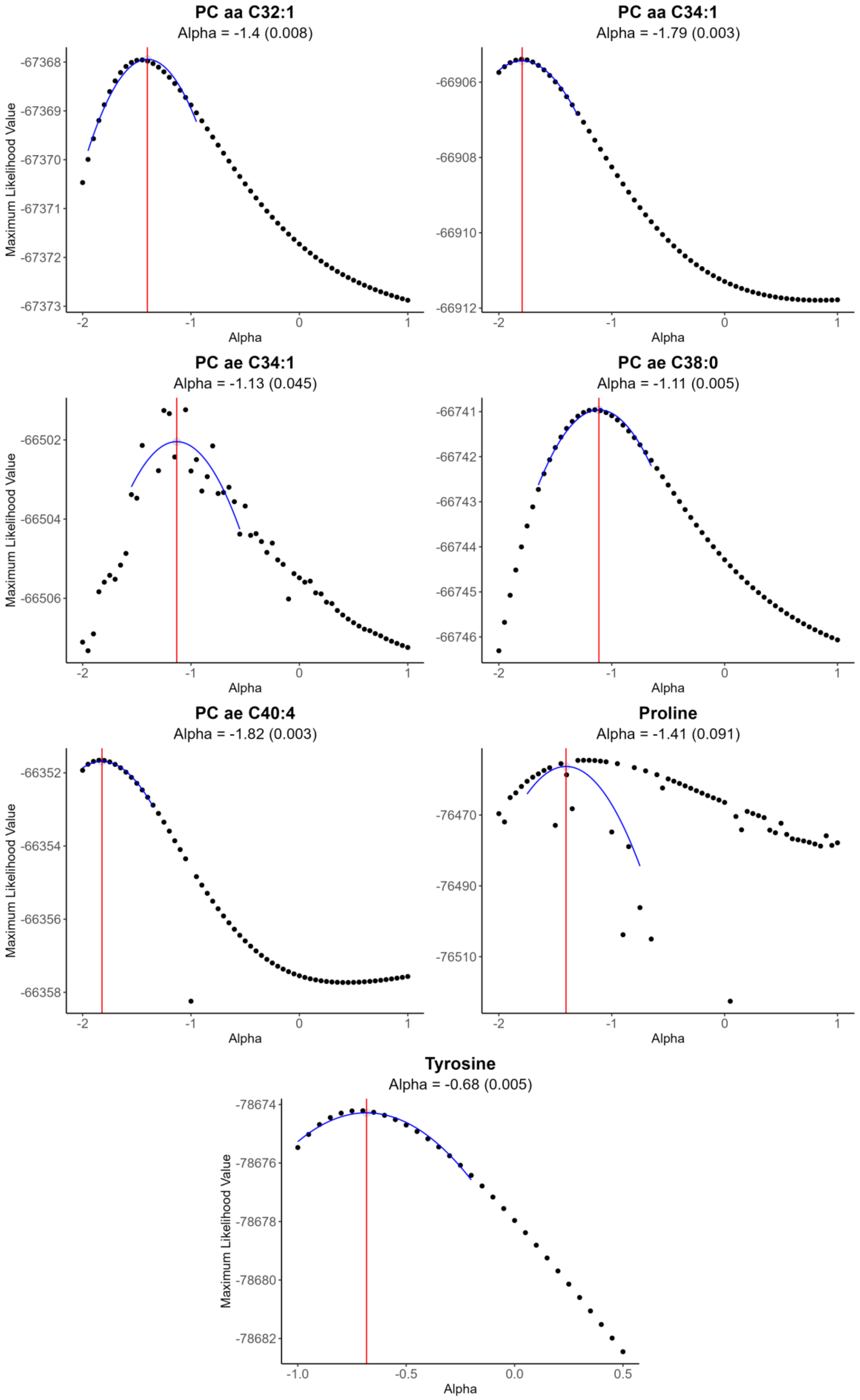

Supplementary Figure S3 (continued). Profile likelihood plots for the metabolites with the selection strength estimates significantly different from 0

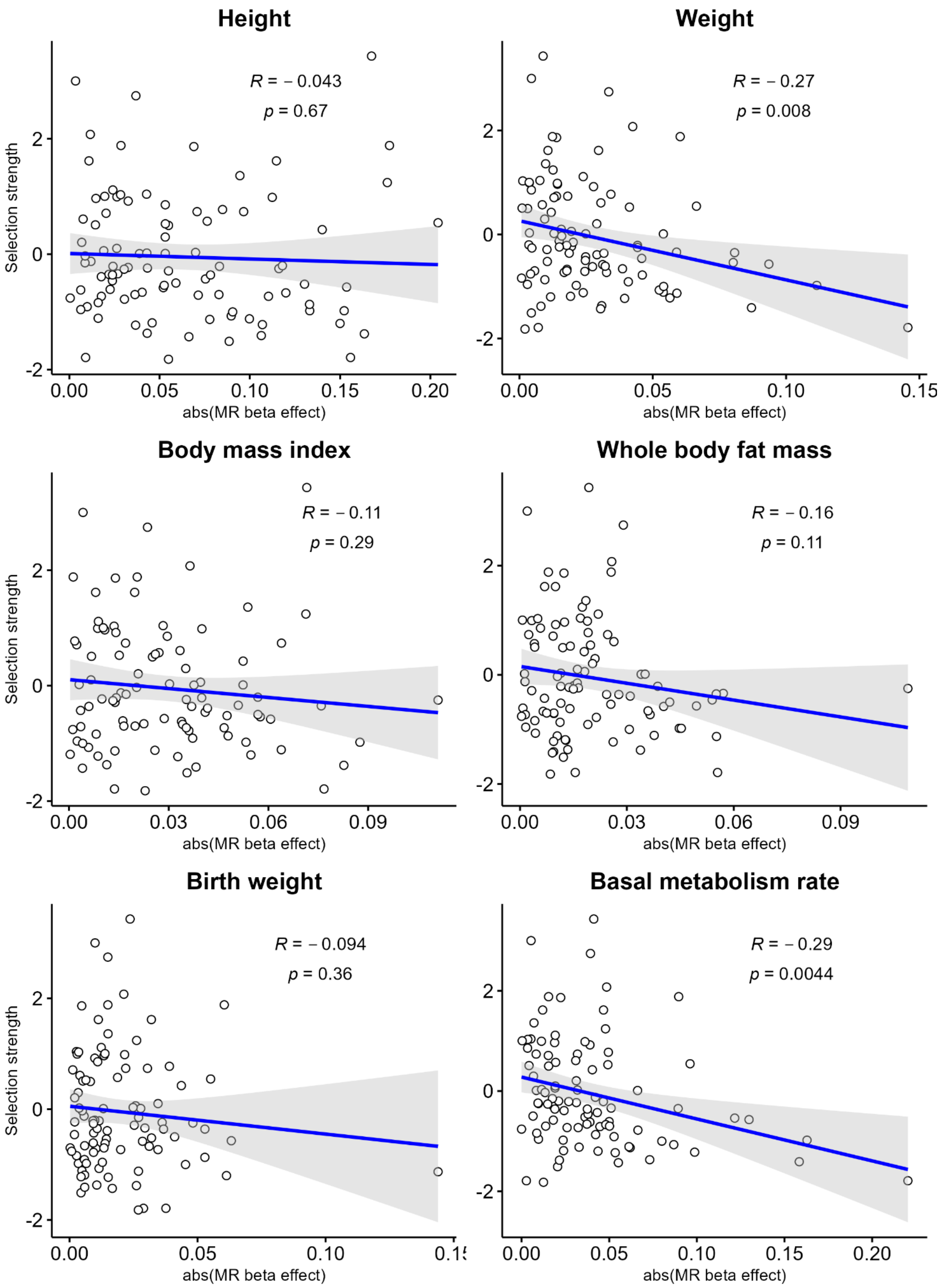

Supplementary Figure S4. Linear relationship between the selection strength estimates and the absolute causal effect sizes obtained by the MR analysis for 51 complex traits using genetic variants associated with the metabolites as instrumental variables.

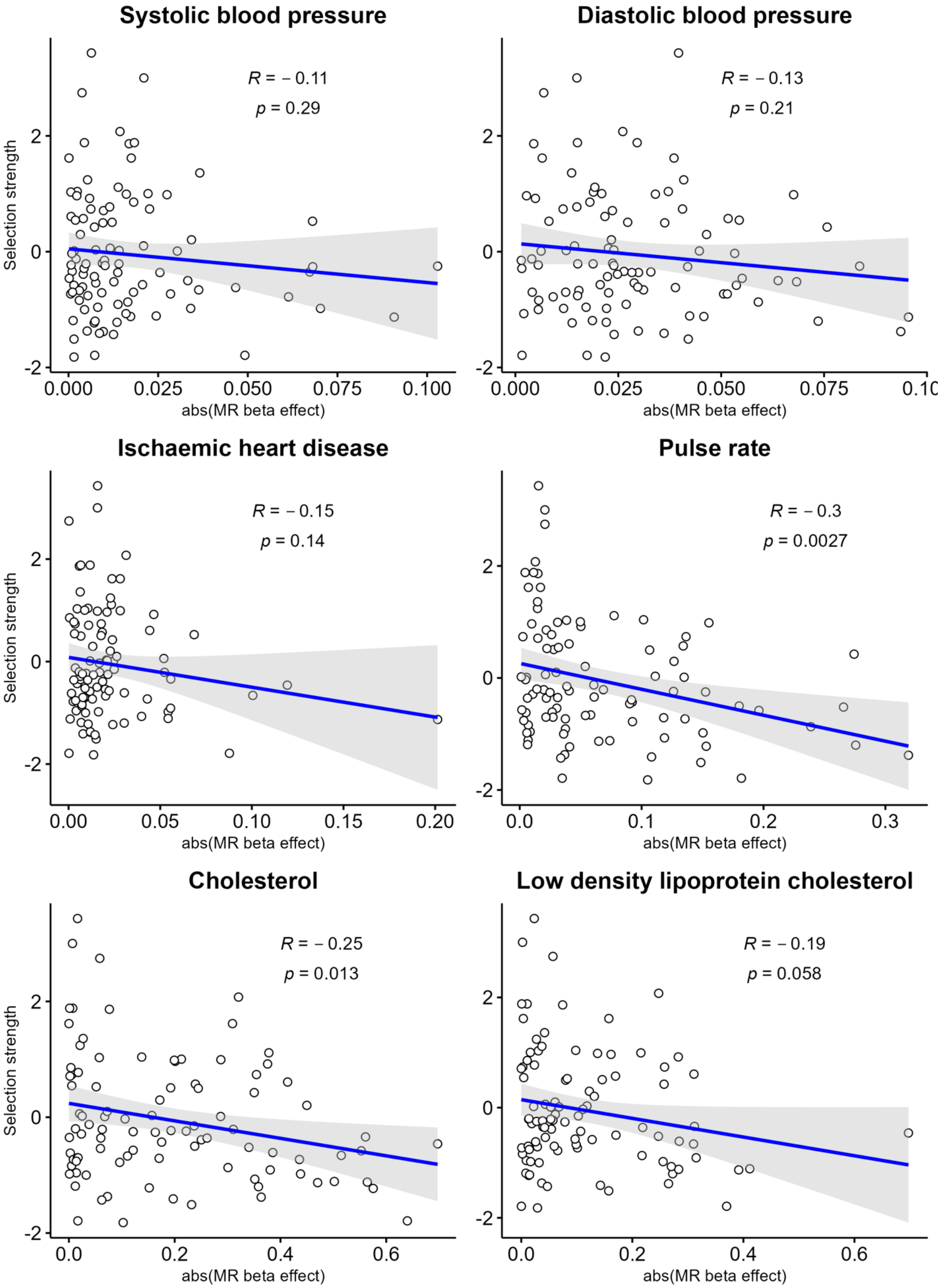

Supplementary Figure S4 (continued). Linear relationship between the selection strength estimates and the absolute causal effect sizes obtained by the MR analysis for 51 complex traits using genetic variants associated with the metabolites as instrumental variables.

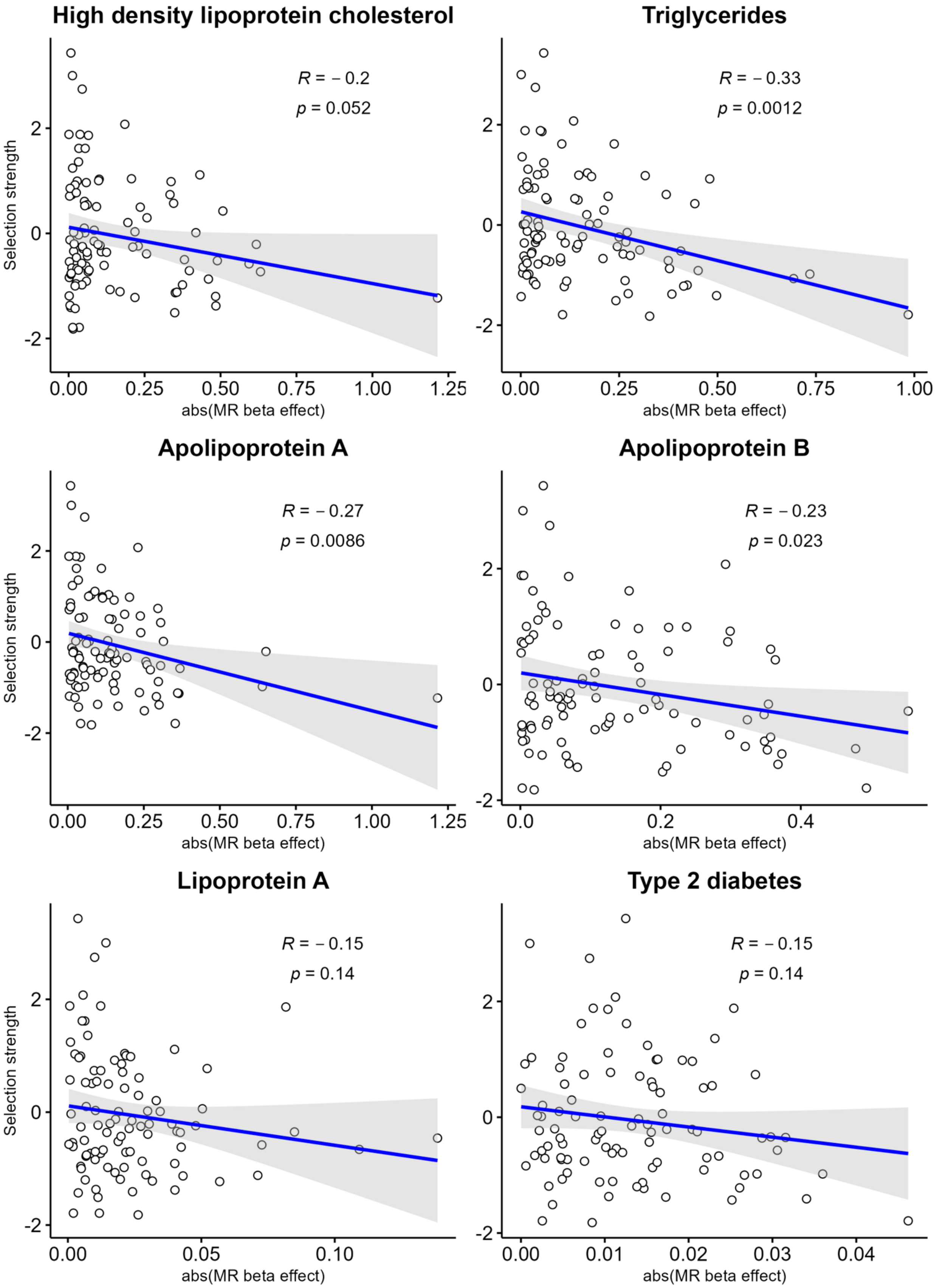

Supplementary Figure S4 (continued). Linear relationship between the selection strength estimates and the absolute causal effect sizes obtained by the MR analysis for 51 complex traits using genetic variants associated with the metabolites as instrumental variables.

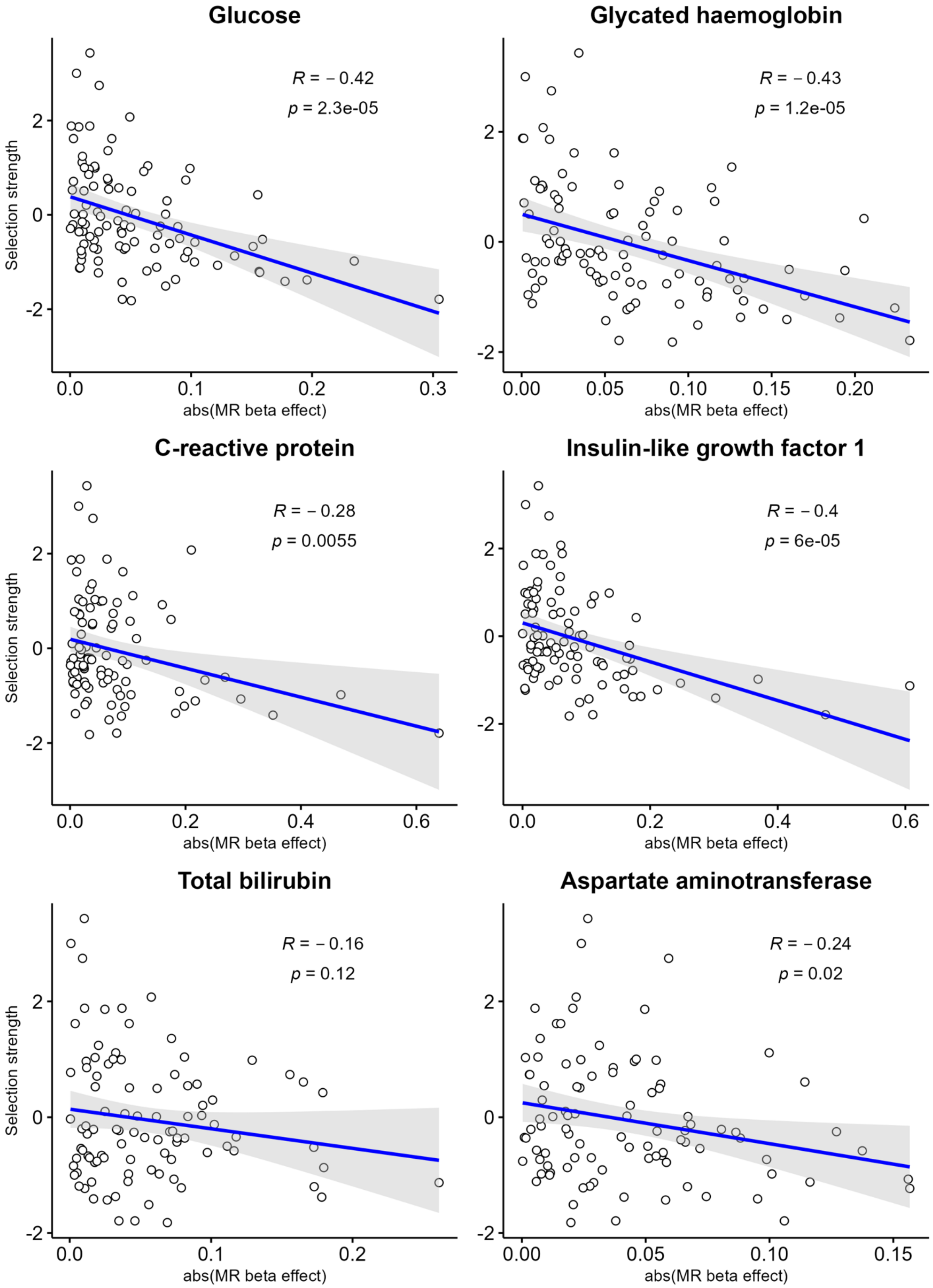

Supplementary Figure S4 (continued). Linear relationship between the selection strength estimates and the absolute causal effect sizes obtained by the MR analysis for 51 complex traits using genetic variants associated with the metabolites as instrumental variables.

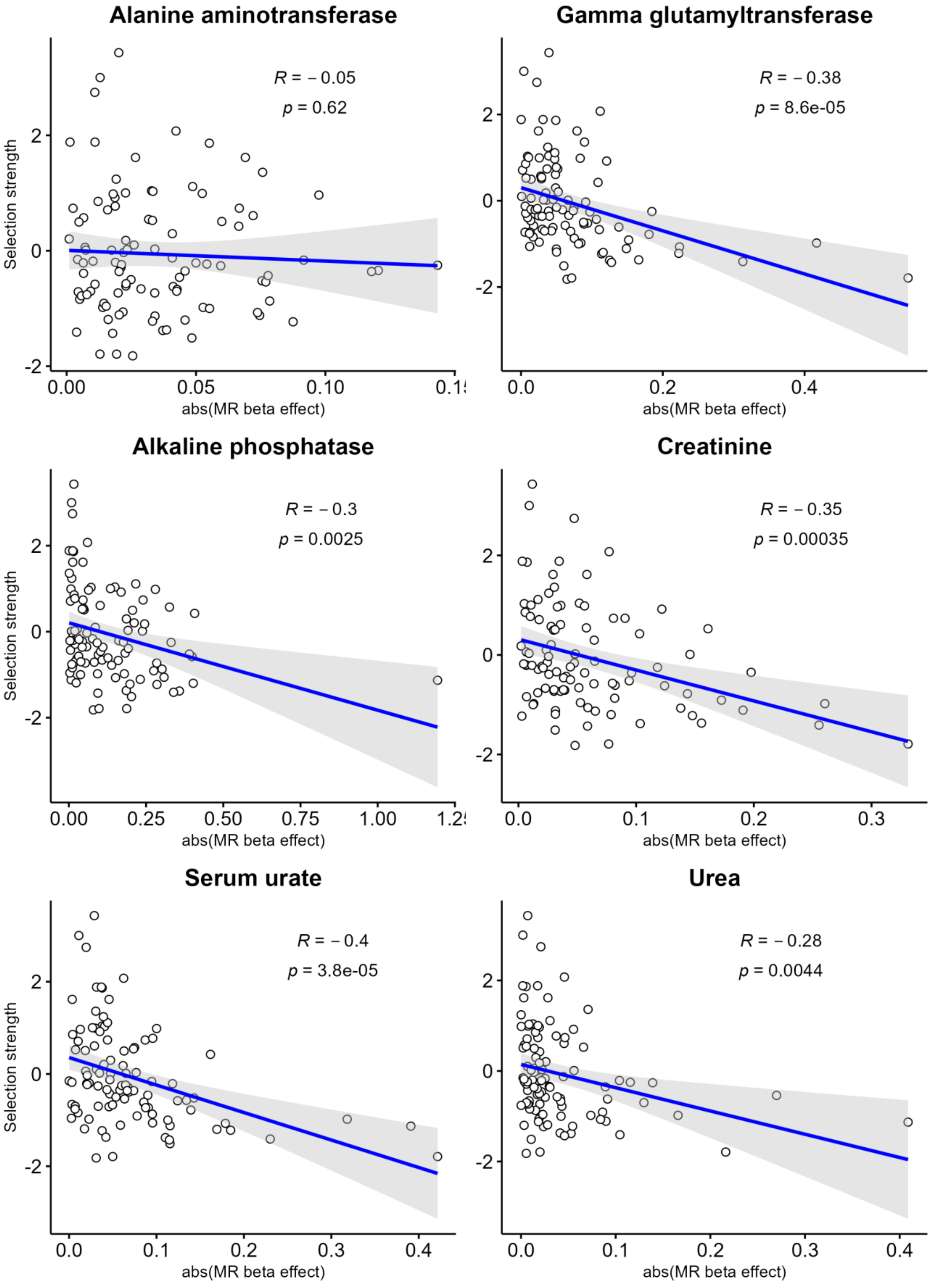

Supplementary Figure S4 (continued). Linear relationship between the selection strength estimates and the absolute causal effect sizes obtained by the MR analysis for 51 complex traits using genetic variants associated with the metabolites as instrumental variables.

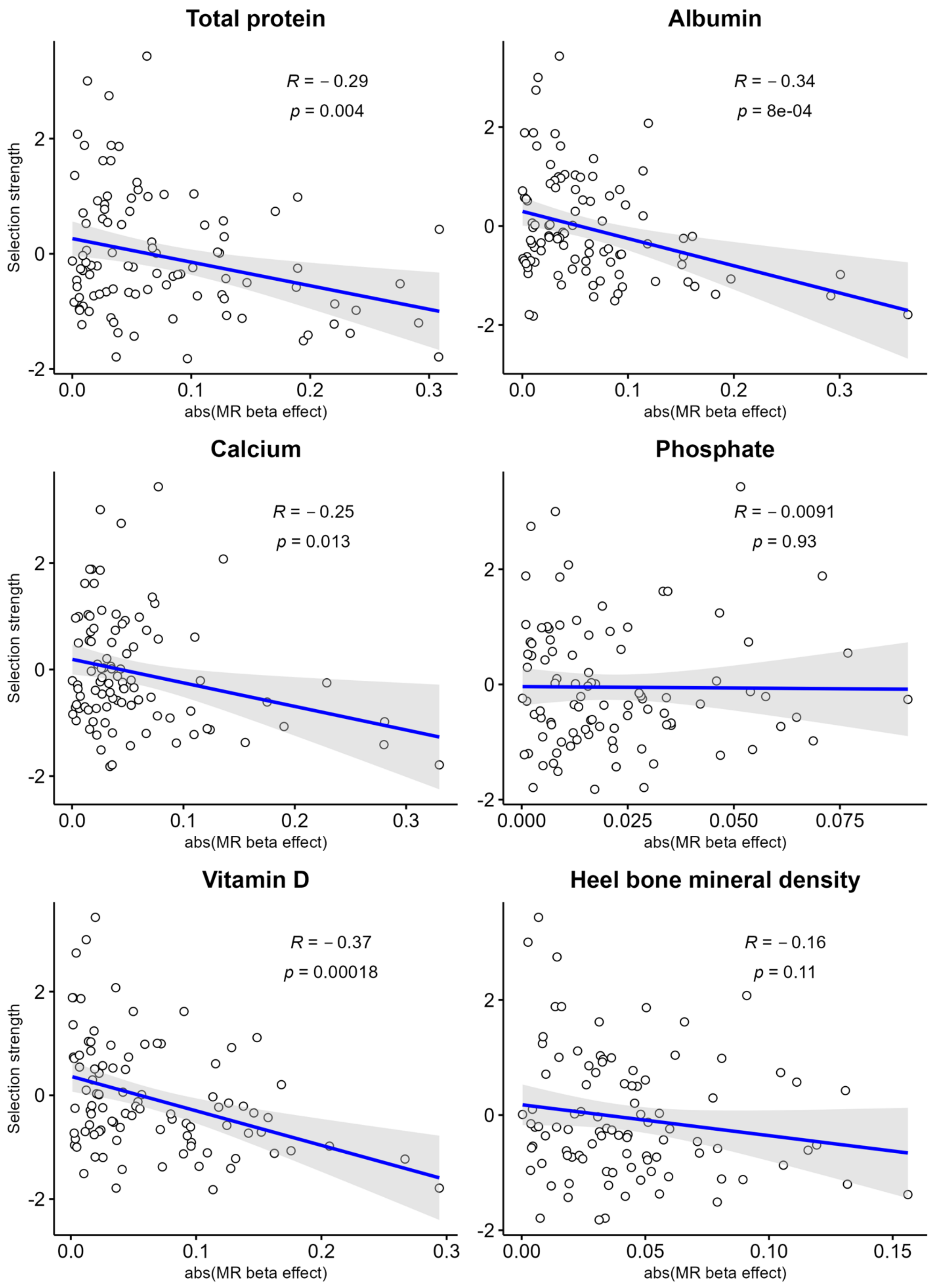

Supplementary Figure S4 (continued). Linear relationship between the selection strength estimates and the absolute causal effect sizes obtained by the MR analysis for 51 complex traits using genetic variants associated with the metabolites as instrumental variables.

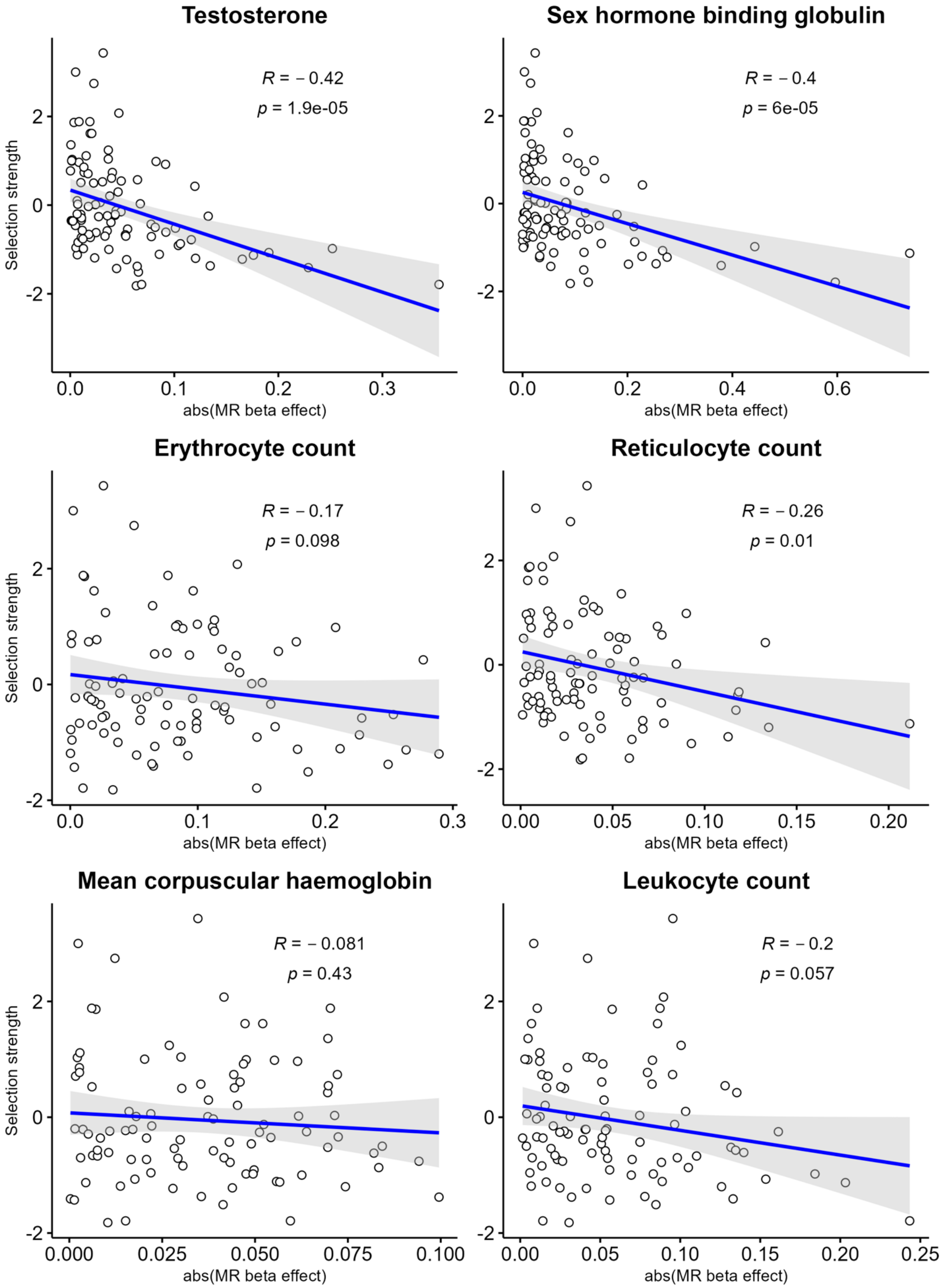

Supplementary Figure S4 (continued). Linear relationship between the selection strength estimates and the absolute causal effect sizes obtained by the MR analysis for 51 complex traits using genetic variants associated with the metabolites as instrumental variables.

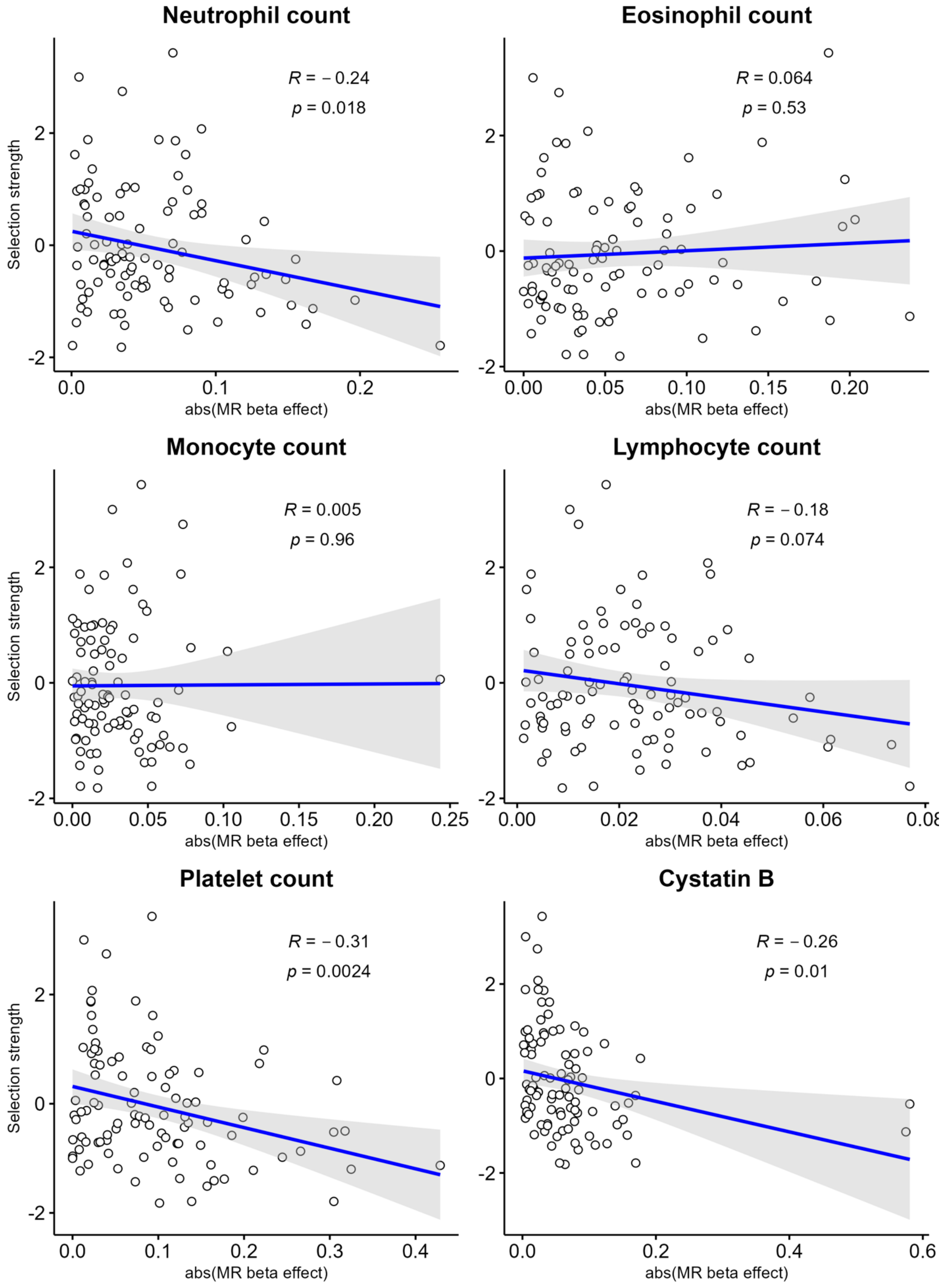

Supplementary Figure S4 (continued). Linear relationship between the selection strength estimates and the absolute causal effect sizes obtained by the MR analysis for 51 complex traits using genetic variants associated with the metabolites as instrumental variables.

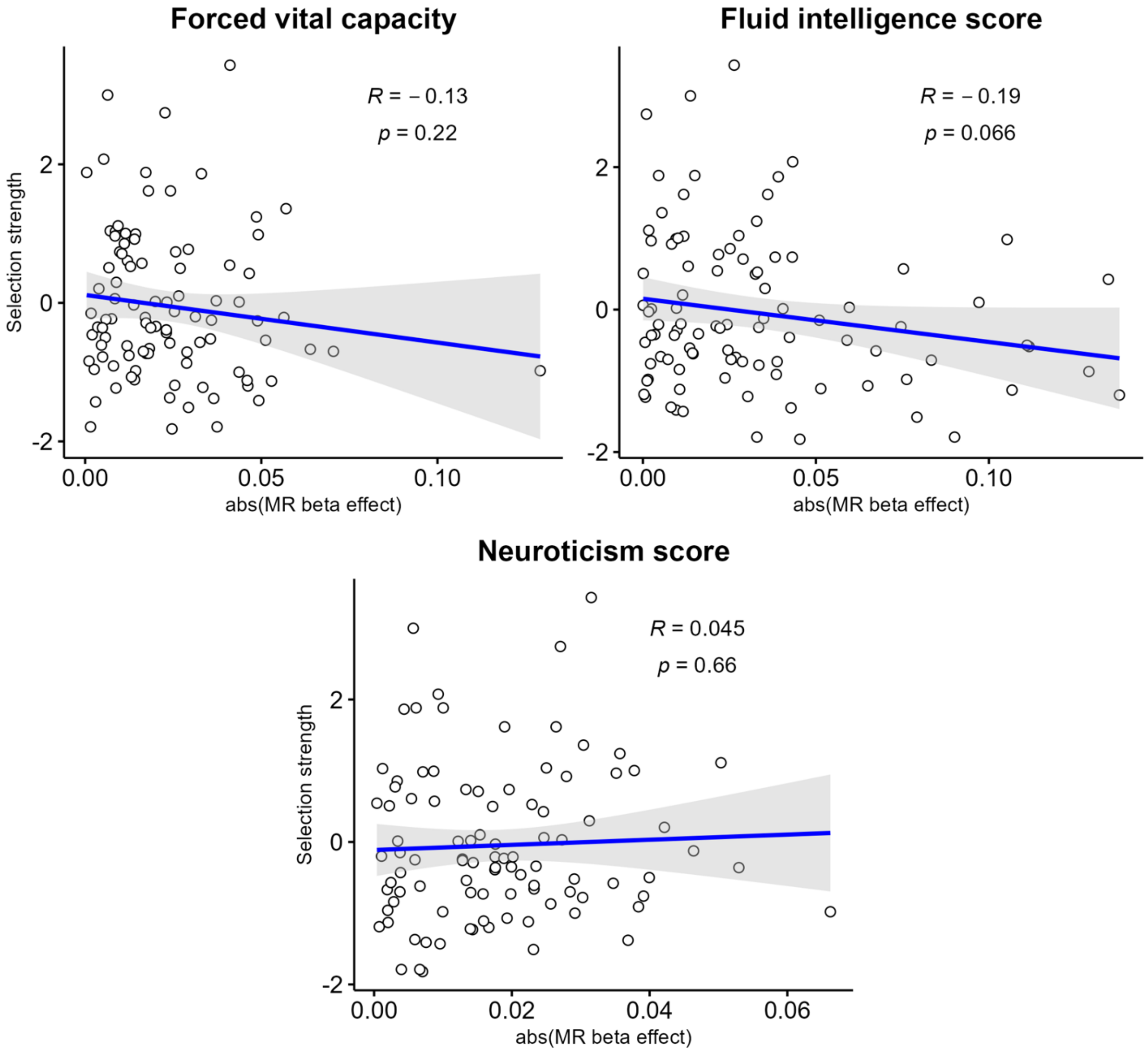

Supplementary Figure S4 (continued). Linear relationship between the selection strength estimates and the absolute causal effect sizes obtained by the MR analysis for 51 complex traits using genetic variants associated with the metabolites as instrumental variables.

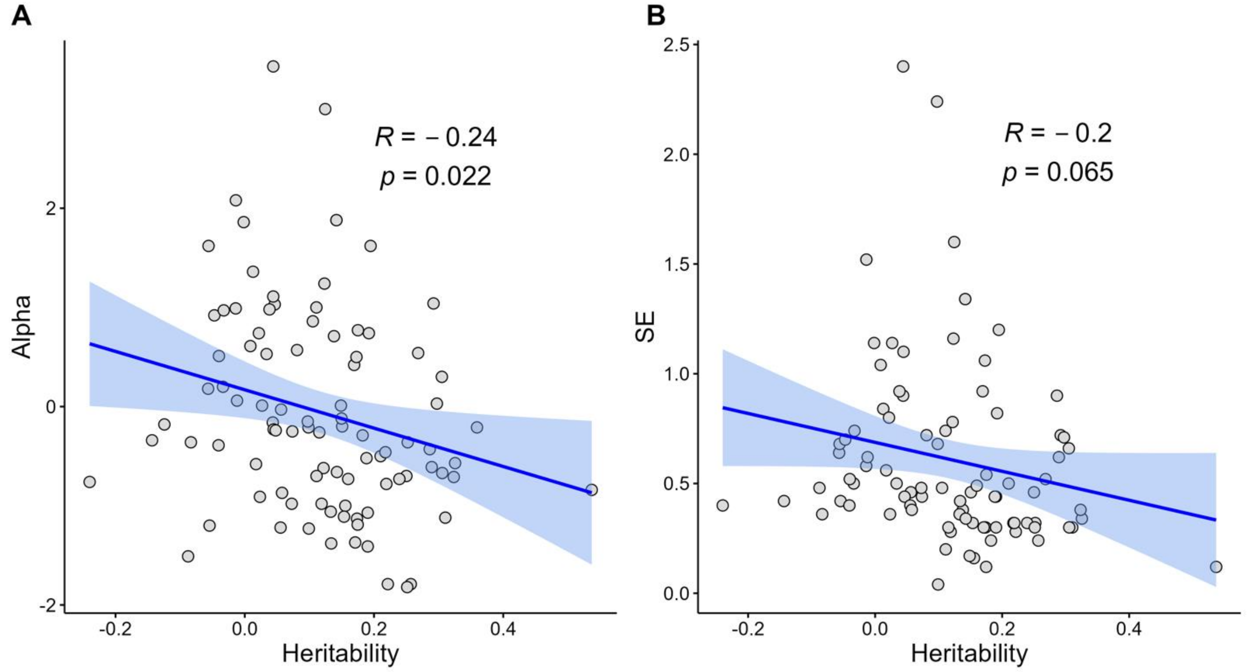

Supplementary Figure S5. Relationship between heritability *vs* selection strength estimate (panel a) / SE of this estimate (panel b).

References:

Schoech AP, Jordan DM, Loh P-R, Gazal S, O'Connor LJ, Balick DJ, Palamara PF, Finucane HK, Sunyaev SR, Price AL. 2019. Quantification of frequency-dependent genetic architectures in 25 UK Biobank traits reveals action of negative selection. *Nature Communications* **10**: 790.

Zeng J, de Vlaming R, Wu Y, Robinson MR, Lloyd-Jones LR, Yengo L, Yap CX, Xue A, Sidorenko J, McRae AF, et al. 2018. Signatures of negative selection in the genetic architecture of human complex traits. *Nature Genetics* **50**: 746-753.
